# Effect of AICAR and 5-Fluorouracil on X-ray Repair, Cross-Complementing Group 1 Expression, and Consequent Cytotoxicity Regulation in Human HCT-116 Colorectal Cancer Cells

**DOI:** 10.3390/ijms18112363

**Published:** 2017-11-08

**Authors:** Ko-Chao Lee, Chien-Tsong Lin, Shun-Fu Chang, Cheng-Nan Chen, Jing-Lan Liu, Wen-Shih Huang

**Affiliations:** 1 Department of Colorectal Surgery, Department of Surgery, Chang Gung Memorial Hospital, Kaohsiung Medical Center, Kaohsiung 833, Taiwan; kmch4329@gmail.com; 2 Center for General Education, National Formosa University, Yunlin 632, Taiwan; mite402@yahoo.com.tw; 3 Department of Wood Based Materials and Design, National Chiayi University, Chiayi 600, Taiwan; 4 Department of Medical Research and Development, Chang Gung Memorial Hospital Chiayi Branch, Chiayi 613, Taiwan; sfchang@cgmh.org.tw; 5 Department of Biochemical Science and Technology, National Chiayi University, Chiayi 600, Taiwan; cnchen@mail.ncyu.edu.tw; 6 Department of Pathology, Chang Gung Memorial Hospital Chiayi Branch, Chiayi 600, Taiwan; b8801046@gmail.com; 7 Graduate Institute of Clinical Medical Sciences, College of Medicine, Chang Gung University, Taoyuan 333, Taiwan; 8 Division of Colon and Rectal Surgery, Department of Surgery, Chang Gung Memorial Hospital, Chiayi 613, Taiwan

**Keywords:** 5-fluorouracil, AICAR, AMP-activated protein kinase, colorectal cancer, X-ray repair cross complementing group 1

## Abstract

Colorectal cancer (CRC) is one of the leading causes of cancer mortality and 5-Fluorouracil (5-FU) is the most common chemotherapy agent of CRC. A high level of X-ray repair cross complementing group 1 (XRCC1) in cancer cells has been associated with the drug resistance occurrence. Moreover, the activation of adenosine monophosphate (AMP)-activated protein kinase (AMPK) has been indicated to regulate the cancer cell survival. Thus, this study was aimed to examine whether XRCC1 plays a role in the 5-FU/AMPK agonist (AICAR)-induced cytotoxic effect on CRC and the underlying mechanisms. Human HCT-116 colorectal cells were used in this study. It was shown that 5-FU increases the XRCC1 expression in HCT-116 cells and then affects the cell survival through CXCR4/Akt signaling. Moreover, 5-FU combined with AICAR further result in more survival inhibition in HCT-116 cells, accompanied with reduced CXCR4/Akt signaling activity and XRCC1 expression. These results elucidate the role and mechanism of XRCC1 in the drug resistance of HCT-116 cells to 5-FU. We also demonstrate the synergistic inhibitory effect of AMPK on 5-FU-inhibited HCT-116 cell survival under the 5-FU and AICAR co-treatment. Thus, our findings may provide a new notion for the future drug regimen incorporating 5-FU and AMPK agonists for the CRC treatment.

## 1. Introduction

Colorectal cancer (CRC) is the third most commonly diagnosed cancer and the third leading cause of cancer death in both men and women [1]. Despite large-scale screening efforts and improved detection and treatment, the overall survival rate of significant numbers of patients with CRC still remains low [2]. Thus far, 5-fluorouracil (5-FU), also used in the treatment of breast cancer, gastric cancer, and other solid tumors, remains the cornerstone of systemic chemotherapy as a first-line treatment in patients with CRC. 5-FU-based chemotherapies are used either alone or in combination with topoisomerase I inhibitor, anti-angiogenic agents, or anti-epidermal growth factor agents. It is well established that treatment of cells with 5-FU and its active metabolite fluorodeoxyuridine monophosphate (FdUMP) causes DNA damage, which in turn influences cell proliferation and survival [3]. In spite of the fact that incidence rates of CRC have declined modestly in recent years, drug resistance to the anti-tumor drugs of cancer cells remains a significant limitation to the clinical use of 5-FU [4]. Strategies for efficacious novel treatment need to be developed to improve the cancer cell response to chemotherapeutic agents.

The base excision repair (BER) pathway in mammalian cells requires four or five steps starting with DNA damage detection and followed by end processing, gap filling, and DNA ligation. Several BER pathway genes play a major role in carcinogenesis and chemotherapy resistance [5]. The X-ray repair, cross-complementing group 1 (XRCC1) plays a central role in the BER system. It facilitates the efficient repair of DNA single-strand breaks (SSBs) and serves as a platform protein and key factor interacting with and modulating the activity of the BER pathway [6]. It has been reported that in the S phase of the cell cycle, XRCC1 played a critical role in DNA replication initiation and replication-coupled repair [7]. If DNA damages are not repaired properly, SSBs may result in genetic instability and eventually generate DNA double-strand breaks (DSBs) during the cell cycle, as well as subsequent tumorigenesis [8]. In addition, an abundant level of XRCC1 transcription has been reported to decrease the cytotoxicity of cisplatin in non-small cell lung cancer [9]. Furthermore, the downregulation of XRCC1 expression resulted in increased sensitivity to the DNA damaging agent methyl methanesulfonate and decreased the SSB repair capacity in human breast cancer cells [10]. Transcriptional regulation of XRCC1 has been reported to be regulated by multiple signaling pathways, such as mitogen-activating protein kinases (MAPKs) or PI3K/Akt [11]. However, whether downregulation of XRCC1 expression is involved in 5-FU-induced cytotoxicity in CRC cells is still unclear.

Deregulation of cellular energetics is a major hallmark of malignant tumor cells [12]. AMP-activated protein kinase (AMPK), activated in response to an increased cellular AMP/ATP ratio, is a crucial cellular sensor in maintaining energy homeostasis [13]. AMPK has been reported to play a role in linking cellular metabolism and tumor suppression by modulating energy levels and inhibiting cancer cell proliferation [14]. There is growing evidence demonstrating the tumor suppressor function of AMPK in CRC and other types of cancer [14]. Previous studies have reported that inflammatory mediators and metabolic components significantly contribute to tumor progression in *CRC,* suggesting AMPK activation may have potential chemoprotective and treatment roles in CRC management [15]. Treatment of human cancer cells with 5-aminoimidazole-4-carboxamide ribonucleotide (AICAR), the pharmacologic activator of AMPK, has been reported to inhibit cell proliferation and induce apoptosis by several mechanisms, including modulating the MAPK and the PI3K/Akt pathways [15]. In addition, AICAR was found to sensitize human CRC cells to death receptor-mediated cytotoxicity through the AMPK signaling pathway in CRC and gastric cancer cells [16,17,18]. These findings suggest that AMPK activation may be used beneficially, alone or combined with chemotherapies, for CRC treatment.

Recent studies have indicated an important role for the CXC chemokine receptor (CXCR4) in regulating the expression of genes involved in tumor progression, angiogenesis, and the metastasis of tumor cells [19]. The activation of CXCR4 and its cognate ligand stromal cell-derived factor-1 leads to the promotion of cancer cell proliferation and migration [20]. Furthermore, increased expression of CXCR4 in human cancer cells indicates that CXCR4 is critical for resistance to chemotherapy. Previous studies suggested that CXCR4 induces chemotherapy resistance in several types of tumors [19,21]. However, the role of CXCR4 in the development of acquired chemoresistance against 5-FU in CRC has not yet been observed.

In the present study, we showed that the expression of CXCR4 and XRCC1 was upregulated in CRC HCT-116 cells treated with 5-FU. We further found that the induction of XRCC1 expression by 5-FU was mediated via the upregulation of CXCR4 expression and the phosphorylation of Akt. Furthermore, AICAR attenuated the 5-FU-induced Akt phosphorylation and XRCC1 expression. These findings on the mechanisms of the suppression of 5-FU-induced responses in CRC cells by AICAR provide new insights into the role of CXCR4 upon the upregulation of XRCC1, and provide potential chemotherapeutic targets in CRC.

## 2. Results

### 2.1. XRCC1 Expression Induced by 5-FU Is Dose- and Time-Dependent in HCT-116 Cells

**Figure 1:**
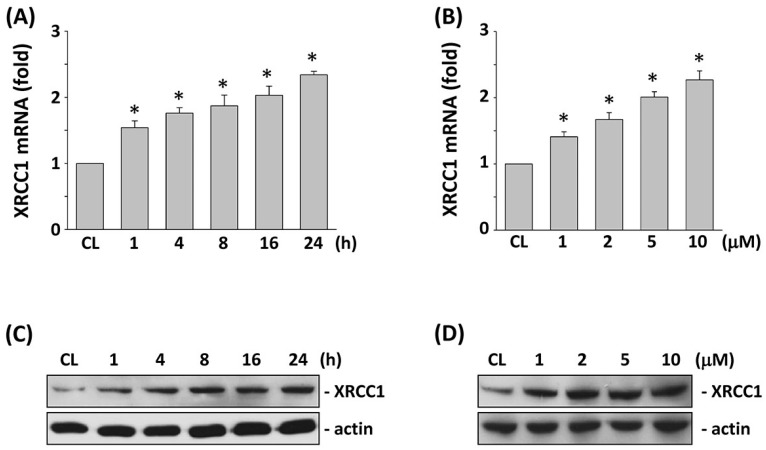
Stimulation with 5-FU increased XRCC1 mRNA and protein levels in HCT-116 cells. HCT-116 cells were kept as controls (CL) or stimulated with 5 μM 5-FU at the indicated time periods (**A**,**C**), or stimulated with different doses of 5-FU for 24 h (**B**,**D**). (**A**,**B**) mRNA expressions of XRCC1 were determined by real-time polymerase chain reaction (PCR) analysis and normalized to 18S rRNA. The results are shown as mean ± standard error of the mean (SEM). * *p* < 0.05 versus CL. (**C**,**D**) XRCC1 protein expressions were determined by Western blot analysis.

To study the effects of 5-FU on XRCC1 expression in CRC cells, HCT-116 cells were used as a cell model. Cells were kept as control or stimulated with 5-FU (5 μM) for the times indicated, or different doses (0, 1, 2, 5, and 10 μM) for 24 h. The changes in mRNA and protein expression of XRCC1 were analyzed by real-time PCR and Western blotting, respectively. The XRCC1 mRNA level began to increase after 1 h of 5-FU stimulation and continued to its highest level at 24 h (Figure 1A). The XRCC1 protein expression also increased after 1 h of stimulation (Figure 1C). In addition, the induction of XRCC1 mRNA and protein expression by 5-FU was in a dose-dependent manner (Figure 1B,D).

### 2.2. Gene Knockdown of XRCC1 in HCT-116 Cells Enhances the Cytotoxicity Induced by 5-FU

**Figure 2:**
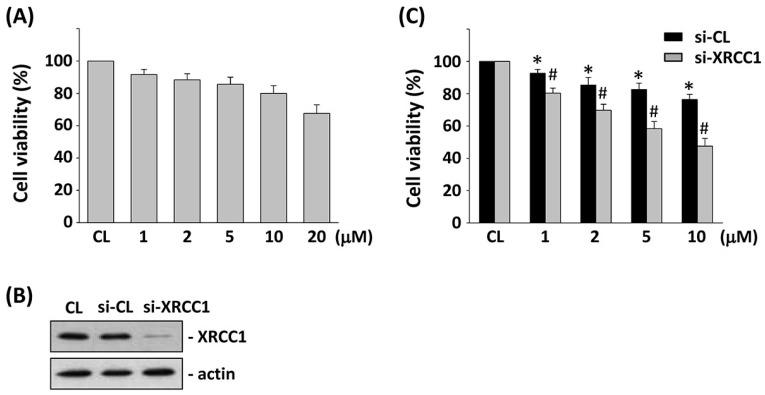
Effects of XRCC1 on 5-FU-induced cytotoxicity in HCT-116 cells. (**A**) HCT-116 cells were kept as controls (CL) or stimulated with different doses of 5-FU for 24 h; cell viability was assayed by the 3-(4,5-dimethylthiazol-2-yl)-2,5-diphenyltetrazolium bromide (MTT) analysis. (**B**) Cells were transfected with control siRNA (si-CL), or a specific siRNA of XRCC1, and then stimulated with different doses of 5-FU for 24 h. Cell viability was assayed by the MTT test. The results are shown as mean ± SEM. * *p* < 0.05 versus CL. ^#^ *p* < 0.05 versus si-CL-treated cells with 5-FU treatment. (**C**) The gene silencing efficiency of 48 h transfection of siRNA on XRCC1 levels of HCT-116 cells was isolated and Western blotting was used to analyze the XRCC1 expression.

To evaluate the effect of 5-FU on HCT-116 cell survival, HCT-116 cells were kept as control or treated with different doses of 5-FU (0–20 μM) for 24 h and analyzed by the MTT assay. Cells stimulated with 5-FU increased cytotoxicity of HCT-116 cells in a dose-dependent manner (Figure 2A). To investigate the role of XRCC1 in the cell viability of CRC cells, we knocked down the XRCC1 expression by using XRCC1-specific siRNA. 5-FU-induced cell cytotoxicity was significantly enhanced by HCT-116 cells transfected with XRCC1 siRNA, suggesting a direct involvement of XRCC1 in the regulation of the cell cytotoxicity of CRC cells against 5-FU stimulation (Figure 2B). The effectiveness of the gene silencing was validated because XRCC1 siRNA (compared with control siRNA) caused a 90% reduction in XRCC1 protein expression (Figure 2C, 25 nM).

### 2.3. PI3K/Akt Signaling Regulates the XRCC1 Expression and Cell Cytotoxicity of HCT-116 Cells under 5-FU Stimulation

**Figure 3:**
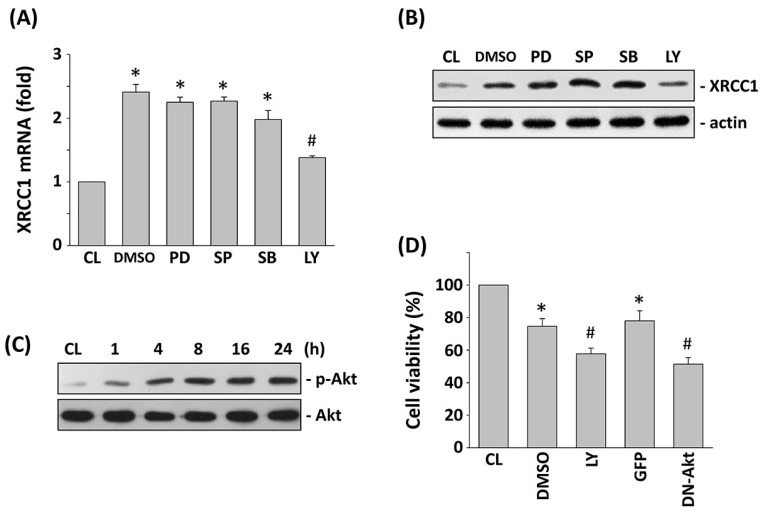
Stimulation with 5-FU regulated XRCC1 expression through Akt activation. HCT-116 cells were pretreated with mitogen-activating protein kinases (MAPK) inhibitor or PI3K/Akt inhibitor individually for 1 h, or infected with vectors expressing the green fluorescent protein (GFP) or dominant-negative (DN)-Akt, and then treated with 5-FU for 24 h (**A**,**B**,**D**) or for indicated time periods (**C**). (**A**) Expression of the XRCC1 mRNA was determined by real-time PCR analysis and normalized to 18S rRNA. (**B**,**C**) The expression of XRCC1 (**B**) and phosphorylation of Akt (**C**) was determined by Western blotting. (**D**) Cell viability was assayed using the MTT test. The results are shown as mean ± SEM. * *p* < 0.05 versus CL. ^#^ *p* < 0.05 versus dimethylsulfoxide (DMSO)- or GFP-treated cells with 5-FU treatment.

To determine whether 5-FU-induced XRCC1 expression and cell cytotoxicity are mediated through the MAPK- or PI3K/Akt-dependent pathways, HCT-116 cells were incubated with specific inhibitors for ERK (PD98059, 30 mM), JNK (SP600125, 20 mM), p38 (SB203580, 10 mM), or PI3K/Akt (LY294002, 20 mM) for 1 h before stimulation with 5-FU. The 5-FU-induced mRNA (Figure 3A) and protein expression (Figure 3B) of XRCC1 was found to be significantly inhibited by LY294002 but not by PD98059, SP600125, and SB203580. The phosphorylation of Akt in HCT-116 cells increased in a time-dependent manner after 1 h of 5-FU stimulation (Figure 3C). To further confirm the involvement of Akt in the modulation of cell cytotoxicity by 5-FU stimulation, we examined the effects of cells pretreated with LY294002 or transfected with dominant-negative (DN)-Akt plasmid on 5-FU-induced cell cytotoxicity. Cell viability was significantly decreased by HCT-116 cells pretreated with LY294002 or transfected with DN-Akt plasmid (Figure 3D).

### 2.4. CXCR4 Regulates the 5-FU Effects on Akt Phosphorylation, XRCC1 Expression, and Cytotoxicity in HCT-116 Cells

**Figure 4:**
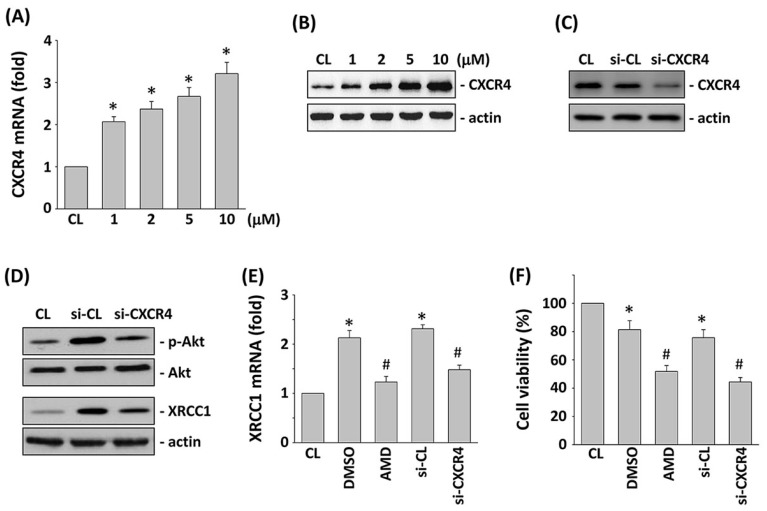
CXCR4 is required for 5-FU-induced Akt phosphorylation and XRCC1 expression. (**A**,**B**) HCT-116 cells were kept as CL or treated with 5-FU for 24 h. mRNA (**A**) and protein (**B**) expression of CXCR4 were determined by real-time PCR and Western blotting analysis, respectively. (**C**) Western blotting was used to analyze the gene silencing efficiency of 48-h transfection of siRNA on the CXCR4 levels of HCT-116 cells. (**D**–**F**) HCT-116 cells were pretreated with CXCR4 inhibitor, or transfected with si-CL or si-CXCR4, and then treated with 5-FU for 24 h. (**D**) The expression of XRCC1 and phosphorylation of Akt was determined by Western blotting. (**E**) mRNA expressions of XRCC1 were determined by real-time PCR analysis. (**F**) Cell viability was assayed by the MTT test. * *p* < 0.05 versus CL. ^#^ *p* < 0.05 versus DMSO-or si-CL-treated cells with 5-FU treatment.

HCT-116 cells were stimulated using different concentrations of 5-FU for 24 h, and the mRNA and protein expression of CXCR4 were analyzed by real-time PCR and Western blotting, respectively. As shown in Figure 4A,B, the CXCR4 mRNA (Figure 4A) and protein (Figure 4B) expression level was increased in a dose-dependent manner. To evaluate the role of CXCR4 in 5-FU-induced Akt phosphorylation and XRCC1 expression, HCT-116 cells were transfected with CXCR4-specific siRNA and followed by stimulation with 5-FU. The CXCR4-specific siRNA (compared with the control siRNA) caused an 80% reduction in CXCR4 protein expression (Figure 4C). The 5-FU-induced Akt phosphorylation and XRCC1 protein expression were significantly suppressed by the gene knockdown of CXCR4 in HCT-116 cells (Figure 4D). To further investigate the role of CXCR4 in the regulation of XRCC1 mRNA expression and cell cytotoxicity by 5-FU stimulation, HCT-116 cells were pretreated with CXCR4-specific inhibitor, AMD3100, or transfected with siRNA against CXCR4 in 5-FU-stimulated cells. The 5-FU-induced XRCC1 mRNA expression (Figure 4E) was significantly inhibited in HCT-116 cells pretreated with AMD3100 or transfected with CXCR4 siRNA. In addition, cell viability was also significantly decreased in HCT-116 cells pretreated with AMD3100 or transfected with CXCR4 siRNA (Figure 4F).

### 2.5. AICAR Regulates 5-FU-Induced XRCC1 Expression and Cell Cytotoxicity

**Figure 5:**
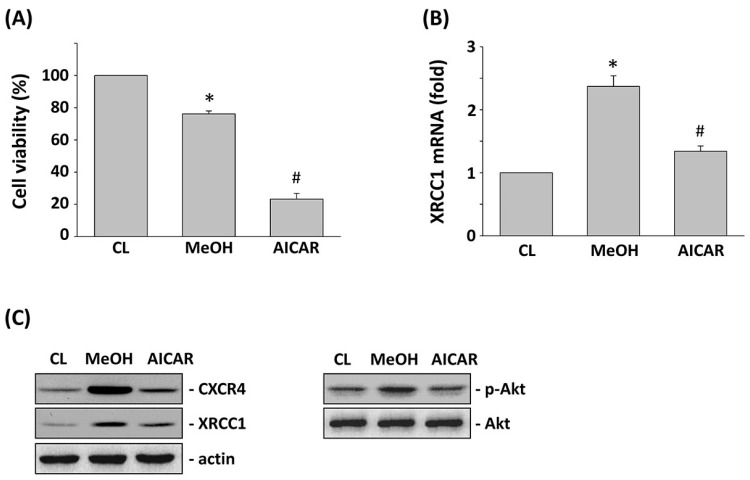
Activated protein kinase (AMPK) agonist 5-FU/AMPK agonist (AICAR) increased 5-FU-induced cytotoxicity, decreased CXCR4 and XRCC1 expression, and decreased Akt phosphorylation. HCT-116 cells were kept as CL, or pretreated with AICAR for 1 h, and then stimulated with 5-FU for 24 h. (**A**) Cell viability was assayed by the MTT test. (**B**) mRNA expressions of XRCC1 were determined by real-time PCR analysis and normalized to 18S rRNA. The results are shown as mean ± SEM. * *p* < 0.05 versus CL. ^#^ *p* < 0.05 versus MeOH-pretreated cells with 5-FU stimulation. (**C**) Western blotting was used to determine the protein expression of CXCR4 and XRCC1, as well as the phosphorylation of Akt.

**Figure 6:**
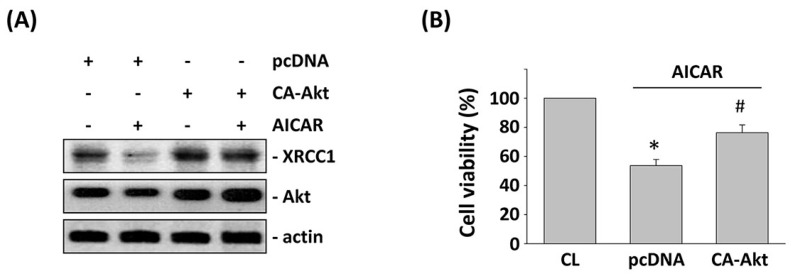
Constitutively active (CA)-Akt regulated 5-FU-induced XRCC1 expression. HCT-116 cells were transfected with pcDNA or constitutively active (CA)-Akt and then treated with AICAR and 5-FU for 24 h. (**A**) Western blotting was used to determine the protein expression of XRCC1 and the phosphorylation of Akt. (**B**) Cell viability was assayed using the MTT test. * *p* < 0.05 versus CL. ^#^ *p* < 0.05 versus pcDNA-transfected cells with AICAR and 5-FU treatment.

AMPK is a potential candidate for cancer therapy. Hence, we investigated the connection between AMPK activation by AICAR and 5-FU-inhibited CRC cell viability. HCT-116 cells were pretreated with AICAR for 1 h and then were stimulated with 5-FU for 24 h. Treating HCT-116 cells with only 5-FU increased the cell cytotoxicity in HCT-116 cells compared with the untreated control. However, pretreating cells with AICAR significantly enhanced the 5-FU effects on the cell viability of HCT-116 cells (Figure 5A). Stimulation of HCT-116 cells with only 5-FU induced XRCC1 mRNA expression compared with the untreated control. However, pretreating cells with AICAR significantly inhibited XRCC1 mRNA expression in HCT-116 cells compared with the 5-FU-only treated cells (Figure 5B). Moreover, pretreating cells with AICAR also significantly inhibited CXCR4 and XRCC1 protein expression and Akt phosphorylation compared with the 5-FU-only treated cells in HCT-116 cells (Figure 5C). We further investigated whether the AICAR-mediated downregulation of XRCC1 was correlated with the regulation of Akt activity in 5-FU-treated HCT-116 cells. Overexpression of the constitutive active (CA)-Akt in HCT-116 cells could rescue the levels of XRCC1 protein and cell viability that were decreased by both of the 5-FU and AICAR treatments in pcDNA-transfected cells (Figure 6A,B).

## 3. Discussion

5-FU is the chemotherapy drug most widely used to kill CRC cells. Unfortunately, cellular drug resistance is the main barrier to chemotherapy, and the identification of the mechanism of 5-FU resistance will substantially improve the anticancer capacity of chemotherapy [22]. This study has provided insights into the mechanism underlying the role of CXCR4 and Akt-mediated XRCC1 expression in affecting 5-FU-induced cytotoxicity. Downregulation of XRCC1 expression potentiated 5-FU-induced cytotoxicity and enhanced its chemosensitizing effect by suppressing CXCR4 and Akt signaling in CRC cells. Our results are significant in several major respects. First, stimulation with 5-FU induces XRCC1 mRNA and protein expression in CRC HCT-116 cells. Second, 5-FU-induced XRCC1 expression is mediated via CXCR4 upregulation and Akt phosphorylation. Finally, activation of AMPK by AICAR enhances the 5-FU effect on cell cytotoxicity by inhibiting Akt activation and XRCC1 expression. Thus, these results elucidate the molecular basis of XRCC1 in the reduction of 5-FU-induced cytotoxicity of CRC cells and the antagonistic role of AICAR in this effect. XRCC1 is a key protein required for DNA SSB repair and genetic stability in cells [23]. A reduction in *XRCC1* expression levels in human breast cancer cells resulted in decreased SSB repair capacity and hypersensitivity to DNA damage induced by methyl methanesulfonate [10]. Previous observation indicated that the expression level of XRCC1 was markedly increased in cisplatin-resistant gastric cancer cells and contributed to cisplatin resistance [24]. In addition, adjuvant platinum-based chemotherapy significantly provides an improved survival benefit in patients with reduced XRCC1 expression in gastric tumoral tissues [25]. Another chemotherapeutic agent irinotecan was also found to decrease the expression of XRCC1 and caused an increase in the sensitivity of cisplatin-resistant cells [24]. In this study, inhibition of XRCC1 expression played a significant role in enhancing the 5-FU-induced cytotoxic effect in CRC cells. The results of this study demonstrate that 5-FU treatment induced XRCC1 mRNA and protein expression in human CRC cells. Our analysis further revealed that the XRCC1 upregulation was mediated via Akt phosphorylation. It has been reported that XRCC1 expression induced by etoposide treatment was dependent on ERK and Akt activation in non-small-cell lung cancer cells [26]. The PI3K/Akt pathway has also been reported to regulate the basal expression of XRCC1 in human tumor cells [27]. The DNA damage and 5-FU-induced SSB can be repaired by BER or other DNA repair proteins, and cell cytotoxicity and drug resistance can be modulated between these DNA repair pathways [11]. Therefore, a mechanism-based approach to increase the 5-FU-induced cell death is to identify how BER DNA repair proteins can contribute to cell cytotoxicity.

Several previous studies have indicated the role of chemokine receptors in tumor progression [27]. Cancer cell CXCR4 expression has been shown to promote tumor progression, suggesting that CXCR4 plays a critical role in the tumorigenesis of CRC [28]. In addition, activation of the Akt pathway is reportedly involved in CXCR4-mediated cell signaling in cancer cells, and this signaling pathway is utilized by CRC cells for invasion, metastasis, and proliferation [29]. CXCR4 expression on tumor cells is indicated to be correlated with a poor prognosis in cancer patients, which may be involved in the chemosensitivity of cancer cells [30]. Here, we showed that CXCR4 regulates 5-FU-induced XRCC1 expression through the activation of Akt. The blockade of CXCR4 affected 5-FU-induced XRCC1 expression and increased cell cytotoxicity. Our results showed activation of Akt following 5-FU stimulation. Cells pretreated with CXCR4 inhibitor AMD3100 or transfected with CXCR4 siRNA demonstrated a blockade of 5-FU-induced Akt phosphorylation. Taken together, these results suggest that Akt activation may be important for 5-FU-induced upregulation of XRCC1. Furthermore, the CXCR4-induced phosphorylation of Akt could lead to the protection of CRC cells against 5-FU-induced cell death.

AMPK, which responds to a variety of metabolic processes, controls cellular nutritional and hormonal signals to maintain energy levels in order to regulate cell growth rate and metabolic homoeostasis [13]. A number of metabolic stresses or pharmacological activators can activate AMPK, and this AMPK in turn regulates various biological processes, including cell proliferation, migration, senescence, and cell death [15]. AICAR, an adenosine analogue, is widely known as an activator of AMPK. AMPK activated by AICAR has been proposed to exhibit an antitumorigenic effect because it is the major signaling network of tumor-suppressor genes, such as LKB1 and TSC1/2 [31]. AMPK activation can result in an anti-tumorigenic effect by inhibition of the metabolic changes that are required for cells to grow rapidly [32]. In addition, previous study has demonstrated that AICAR significantly inhibits cancer cell viability and proliferation without affecting normal cells [33]. AICAR has also been reported to reduce cancer cell growth through blocking the AKT/FOXO3a signaling pathway [34]. It has been reported that AMPK activation and 5-FU synergistically enhanced the antitumor effects of 5-FU on different types of cancer cells [17,18,35]. Another study demonstrated that enhancement of the cytotoxicity to cisplatin by administration of curcumin, an anti-inflammatory molecule in the turmeric root, is mediated by the downregulation of the expression levels of XRCC1 in human lung cancer cells [36]. In this study, inhibition of Akt activation, and CXCR4 and XRCC1 expression by AICAR, enhanced the 5-FU-induced cytotoxicity in CRC cells. The detailed molecular mechanism of AICAR and its combination with 5-FU on cell death in CRC cells were under our investigation.

In summary, the present study demonstrates that AICAR has a synergistic cytotoxic effect with 5-FU in CRC cells through the suppression of CXCR4 and XRCC1. The results of this study suggest that decreasing XRCC1 expression may enhance the therapeutic effect of 5-FU in patients with CRC. However, it has been indicated that the polymorphism of XRCC1 gene, including the Arg194Trp which is a substitution of arginine to tryptophan at position 194 of XRCC1, also plays an important role in affecting the susceptibility, prognosis, and therapy outcome of the clinical CRC patient with platinum/5-FU treatment [37,38]. Thus, further study is needed to investigate the role of XRCC1 gene polymorphism in CRC treated with AICAR, 5-FU, and their combination. Moreover, the in vivo animal studies are also needed to confirm these in vivo studies.

## 4. Materials and Methods

### 4.1. Materials

All culture materials were purchased from Gibco (Grand Island, NY, USA). PD98059 (ERK inhibitor), SP600125 (JNK inhibitor), SB203580 (p38 inhibitor), and LY294002 (PI3K/Akt inhibitor) were purchased from Calbiochem (La Jolla, CA, USA). Mouse monoclonal antibodies (mABs) against Akt and phospho-Akt were purchased from Santa Cruz Biotechnology (Santa Cruz, CA, USA). Rabbit polyclonal antibodies against XRCC1 and mouse monoclonal CXCR4 antibody were purchased from Cell Signaling Technology (Beverly, MA, USA). The CXCR4- and XRCC1-siRNA and control siRNA (scrambled negative control containing random DNA sequences) were purchased from Thermo (Waltham, MA, USA). AICAR and all other chemicals of a reagent grade were obtained from Sigma (St. Louis, MO, USA).

### 4.2. Cell Culture

The colon cancer HCT-116 cell line was purchased from the Bioresources Collection and Research Center (BCRC) of the Food Industry Research and Development Institute (Hsinchu, Taiwan). Cells were maintained in Dulbecco’s Modified Eagle Medium (DMEM) supplemented with 10% fetal bovine serum (FBS) and 1% penicillin/streptomycin in a CO_2_ incubator at 37 °C.

### 4.3. MTT Assay

HCT-116 cells were cultured in 96-well plates. Cell viability was determined by MTT assay. After the incubation period, 3-(4,5-dimethylthiazol-2-yl)-2,5-diphenyltetrazolium bromide (MTT) solution was added to each well to a final concentration of 0.5 mg/mL, and the mixture was incubated at 37 °C for 3 h to allow MTT reduction. The formazan crystals were dissolved by adding dimethylsulfoxide (DMSO). Absorbance was measured at 570 nm with a spectrophotometer.

### 4.4. Real-Time Quantitative PCR

The total RNA was isolated by the guanidium isothiocyanate/phenol/chloroform method and converted to cDNA. Real-time PCR of three transcripts was performed using an ABI Prism 7900HT with the FastStart DNA SYBR Green I kit (Applied Biosystems, Foster City, CA, USA). The design primers in this study were: XRCC1 forward primer, 5′-GTGAC ATGCA GCACC TCCTG-3′; XRCC1 reverse primer, 5′-TCCAT GGTGA TCTCT CCTCA-3′; 18S rRNA forward primer, 5′-CGGCG ACGAC CCATT CGAAC-3′, and 18S rRNA reverse primer, 5′-GAATC GAACC CTGAT TCCCC GTC-3′. Quantification was performed using the 2^−ΔΔ*C*t^ method [39]. The PCR conditions were optimized to obtain a PCR product with a single peak on melting curve analysis. All samples were measured in duplicate. The average value of both duplicates was used as the quantitative value.

### 4.5. Western Blot Analysis

Samples were lysed with a buffer containing 1% NP-40, 0.5% sodium deoxycholate, 0.1% SDS, and a protease inhibitor mixture (PMSF, aprotinin, and sodium orthovanadate). Protein concentration was determined using the Bio-Rad protein assay kit (Bio-Rad, Hercules, CA, USA). Equal amounts of total proteins (70 µg of protein) were separated by SDS-polyacrylamide gel electrophoresis (PAGE) (10% running, 4% stacking), transferred onto a nitrocellulose membrane, blocked with 10% nonfat milk in Tris-buffered saline with Tween 20, and incubated with designated primary antibodies at 4 °C overnight. After washes, the primary antibodies were detected with horseradish peroxidase-conjugated secondary antibodies (incubated for 1 h) and analyzed using the designated antibodies and the Western-Light chemiluminescent detection system (Bio-Rad).

### 4.6. Dominant Negative (DN)-Akt, Constitutively Active (CA)-Akt, and siRNA Transfection

The DN-Akt was kindly provided by Dr. Yi-Shuan Li (University of California, San Diego, CA, USA). For plasmid and siRNA transfection, HCT-116 cells were maintained in DMEM supplemented with 10% FBS in a CO_2_ incubator at 37 °C overnight and then transfected with the plasmid by using an Lipofectamine 2000 transfection reagent (Thermo, Waltham, MA, USA) or transfected with the control-, XRCC1-, or CXCR4-specific siRNA by using an RNAiMAX transfection reagent (Thermo, Waltham, MA, USA). After 48 h incubation, the transfected cells were used in the designated experiments.

### 4.7. Statistical Analysis

The results are expressed as the mean ± standard error of the mean (SEM). Statistical analysis was determined using an independent Student *t*-test for two groups of data and analysis of variance (ANOVA) followed by Scheffe’s test for multiple comparisons. *p* values less than 0.05 were considered significant.
